# The lateral trauma position – what do we know, how do we use it? A cross-sectional survey of all Norwegian emergency medical services

**DOI:** 10.1186/1757-7241-20-S1-O5

**Published:** 2012-03-22

**Authors:** S Fattah, G Ekås, PK Hyldmo, T Wisborg

**Affiliations:** 1 Hammerfest Hospital, Finnmark Health Trust, Norway; 2 Norwegian Air Ambulance Foundation, Department of Research, Norway; 3 Kongsvinger Hospital, Innlandet Health Trust, Norway; 4 Sørlandet Hospital Health Trust, Arendal & Kristiansand, Kristiansand, Norway; 5 University of Tromso, Institute of Clinical Medicine, Tromso, Norway

## Introduction

Traditionally trauma patients have been transported in the supine position to protect the spine. The ABCDE-principle however clearly points out the priority of securing airways. In Norway the lateral trauma position (LTP) has gradually been introduced since 2005. After checking airways and breathing, the unconscious trauma patient is rolled into a lateral (recovery) position, maintaining manual in-line stabilisation with c-collar. Padding is used to obtain neutral spine position.

## Aim

investigate implementation and present use of LTP in Norwegian Emergency Medical Services (EMS).

## Methods

All ground and air EMS bases throughout the country were included. Telephone interviews were performed with ground EMS supervisors. Questionnaires were distributed to ground EMS personnel. Medical supervisors of the air EMS were included. The research protocol was approved by the Regional Ethics Committee.

## Results

Of 202 ground EMS supervisors approached 201 answered, 75% reported that LTP is used in their area. Written protocols were present in 61% of these areas, and 69% have provided training to their employees in LTP use.

Questionnaires were distributed to 3025 ground EMS personnel. We received 1395 (46%) valid answers. LTP was known by 89%, but only 59% answered that they use it. Of the respondents using LTP 77% reported access to written protocols, 78% flex the upper knee, while 20% flex the lower; padding under the head was reported by 81%.

Of 24 air EMS supervisors 24 participated. LTP is used by 52% of the services, although only 8% of these have a written protocol and 25% arrange training in the use of LTP.

## Conclusions

LTP is implemented and used in the majority of the Norwegian EMS, despite little evidence as to its possible benefits and harms. How the patient is positioned in LTP differs. More research on the method is essential to establish its evidence base.

